# Progress in Obstetrics

**Published:** 1900-01-13

**Authors:** 


### PROGRESS IN OBSTETRICS.

(*Continued from page 228.*)

**The Local Treatment of Puerperal Infection.**—Arnold Lea,[12] in a paper on this subject, points out that from a clinical point of view we have no reliable means of ascertaining in the first instance whether we are dealing with a local absorption of soluble toxins—sapræmia—or a general septicæmic condition. Further, the onset of acute pelvic cellulitis or a local inflammatory affection of the appendages is indistinguishable at first from a case of sapræmia or septicæmia. Hence, he maintains that in all cases of infection a similar line of treatment should be adopted at first. From an analysis of 48 cases observed by himself, he draws the following conclusions as regards treatment: (1) A rise of temperature over 101·4 during the puerperium, not obviously accounted for by other causes, should lead to a thorough examination of the genital passage. (2) If no sufficient explanation is found in the condition of the perineum, or vagina, a uterine douche should be at once given with due precautions. (3) If within 24 hours the temperature has fallen definitely, no further exploration is required, but the douche may be repeated if the temperature again rises. (4) If at the end of 24 hours the temperature is higher, and the pulse rate has increased, the cavity of the uterus should be explored with the sterilised finger. (5) If the initial rise of temperature is great (103 deg. or over), with or without a rigor, the uterus should be explored at once, without waiting 24 hours to observe the effect of a douche. This is more especially indicated if the uterus is bulky, showing delayed involution, since this points to putrefaction of retained products or to septic endometritis. (6) If clots or placenta are discovered, they should be removed by the finger or curette, a douche given, and a gauze drain inserted for 24 hours. (7) In the great majority of cases it is wiser to thoroughly curette the uterus with the object of removing the whole of the decidua and retained products. (8) There is no evidence that curettage, if done with every precaution, favours the spread of infection. In a large proportion of cases the infection is rapidly checked. (9) In very virulent infection early curetting, with the object of sterilising the uterine cavity, affords the best chance of a successful result. (10) If curettage entirely fails it must be repeated or not, according to the local condition present. The prognosis, however, in the absence of a definite localisation of the infective process, is bad. (11) In some cases if curettage fails, and there is no evidence of general peritonitis or of infection of the blood serum, vaginal hysterectomy (if performed in good time) may be successful. (12) Antistreptococcic serum should be given early and freely in cases of proved streptococcic infection. It is of little use in the advanced stages of the disease. Dr. Spencer[13] does not expect much benefit from removal of the uterus in puerperal sepsis, and thinks the cases for which it is suitable must be very few. As regards the serum treatment, he says the conclusions to which the study of the subject of serum therapy has led him are: (1) That as usually applied it has not a scientific basis; (2) that it has not lowered the mortality of puerperal sepsis; (3) that it usually lowers the temperature, and sometimes improves the general condition; and (4) that its use is not free from danger. Whether it will prove of value in the treatment of pure streptococcic infection (detected by the examination of the uterus or the blood) remains for the future to show. More Madden[14] points out that it must never be forgotten that so-called "puerperal fever" is not a distinct form of disease due to a specific infection. If it were one might more readily accept the theory of its amenability to a specific serum therapy. These different forms of disease obviously require different treatment, and no one form of antitoxin could be applied to them all. He believes that no modern method has yet superseded the old-fashioned combination of quinine and grey powder, which he had long recommended as the best germicide and antipyretic in these cases.

**Eclampsia.**—Dr. Jardine[15] recommends the intercellular injection of saline fluid in the treatment of eclampsia. He believes the convulsions are probably caused by toxic substances being retained in the blood which should have been excreted by the kidneys. The liver also seemed to be involved in some way. Frieben[16] describes changes in the liver in two fatal cases of eclampsia, consisting of specks of blood under the serous coat, and in the deeper part of the parenchyma, but he believes they are quite secondary, being due to the mechanical effect of the convulsions. The toxic matter, Jardine says, accumulates in the blood, and as the kidneys are unable to accomplish the extra work thrown upon them it so seriously affects the nervous system that an explosion occurs in the form of convulsions. If a toxic substance is circulating through the system it may be got rid of by three main channels—the bowels, the skin, and the kidneys. All of these channels are useful, but the most useful one is that belonging to the system involved. In the case of eclampsia it is through the kidney that we should strive to get rid of the poison, at the same time aiding the result by purgation and diaphoresis. When the fits have occurred purgation and diaphoresis may usually be brought about by means of croton oil, enemata, and a hot pack or bath. It is more difficult, however, to get the kidneys to act. Often the patient is unable to swallow, and if this is possible diuretics by the mouth are slow in action, and time is of the utmost importance. For this end saline injection has been found very useful. The solution used by Dr. Jardine consisted of one part of bicarbonate of potash to three parts of common salt, and of this one drachm to the pint of sterilised water at 100 deg. Fahr. The potash was added owing to its powerful diuretic action,

The injection might be made into any part of the body when the tissues were loose. Under the edge of the breast was a convenient place before delivery, and the lax abdominal wall after. One pint of the solution has been injected in four minutes. Absorption began at once, and it all disappeared in from 15 to 20 minutes. In 12 cases treated in this way the diuretic effect was very marked. For controlling the fits, Jardine recommends the use of chloral, bromide, and veratrum viride, along with the injections. Morphia, he believes, interferes with the action of the kidney. If labour has commenced, he thinks it advisable to deliver as soon as possible. If, on the other hand, labour has not commenced, he is inclined not to interfere unless the fits persist in spite of treatment.

[12] Arnold Lea, Med. Chron., Aug., 1899. [13] Spencer, Brit. Med. Jour., Oct., 1899. [14] More Madden, Lancet, Aug. 12, 1899. [15] Jardine, Scottish Med. Jour., Oct., 1899. [16] Frieben, Brit. Med. Jour., July 15, 1899.

